# Sustainable Removal of BTEX Gas Using Regenerated Metal Containing SiO_2_

**DOI:** 10.3390/nano12234113

**Published:** 2022-11-22

**Authors:** Soyoung Beak, Yasaman Ghaffari, Suho Kim, Eun Ji Kim, Kwang Soo Kim, Jiyeol Bae

**Affiliations:** 1 Department of Environmental Research, Korea Institute of Civil Engineering and Building Technology (KICT), Goyang 10223, Republic of Korea; 2 Civil and Environmental Engineering, University of Science and Technology (UST), Daejeon 34113, Republic of Korea; 3 Department of Civil & Environmental Engineering, Yonsei University, Seoul 03722, Republic of Korea

**Keywords:** BTEX removal, UV-assisted regeneration, nanocomposites, metal oxide, reusability

## Abstract

In the last decades, the removal of benzene, toluene, ethylbenzene, and xylene (BTEX) has been considered a major environmental crisis. In this study, two novel nanocomposite materials (Fe_2_O_3_/SiO_2_ and Fe_2_O_3_-Mn_2_O_3_/SiO_2_) that have regeneration ability by UV irradiation have been fabricated to remove BTEX at ambient temperature. This research revealed that both nanocomposites could remove more than 85% of the BTEX in the first cycle. The adsorption capacities followed the order of ethylbenzene > m-xylene > toluene > benzene as in the molecular weight order. The reusability test using UV irradiation showed that the performance of Fe_2_O_3_/SiO_2_ decreased drastically after the fifth cycle for benzene. On the other hand, when Mn is located in the nanocomposite structure, Fe_2_O_3_-Mn_2_O_3_/SiO_2_ could maintain its adsorption performance with more than 80% removal efficiency for all the BTEX for ten consecutive cycles. The difference in the reusability of the two nanocomposites is that the electron energy (from the valence band to the conduction band) for BTEX decomposition is changed due to the presence of manganese. This study provides a promising approach for designing an economical reusable nanomaterial, which can be used for VOC-contaminated indoor air.

## 1. Introduction

Volatile organic compounds (VOCs) are recently considered to be a major cause of air pollution and harm to health [1,2,3]. Catalytic oxidation is one of the most useful approaches toward VOC removal from industrial sources and indoor/outdoor environments [4,5]. Benzene, toluene, ethylbenzene, and xylenes (o-, m- and p-) (BTEX) are the main representatives of VOCs. They have carcinogenic and mutagenic effects. Exposure to BTEX is associated with adverse effects on the liver, nervous system, heart, and kidneys and increases the risk of developing nonlymphocytic leukemia [6,7,8]. In recent years, different nanomaterials such as metal–organic framework (MOF) [9], covalent organic framework (COF) [10], zeolitic–imidazolate framework (ZIF) [11], TiO_2_ [12], and perovskites [13] has been used for VOC removal. However, in most cases, the techniques had several drawbacks, such as high cost, and low efficiency. Moreover, these methods could not be reused for real conditions. 

Yang et al. investigated the adsorption of o-xylene, m-xylene, p-xylene, and ethylbenzene in the air on a synthesized MOF-177 and reported that the adsorption of VOCs on MOF-177 in humid air was suppressed by the competition of water molecules and the decomposition of the MOF-177 framework structure with water [14]. Aziz et al. reported VOCs removal on Fe-loaded-ZSM-5, wherein removal performance decreased significantly after the second cycle and the material showed very poor performance (<30%) in the removal of benzene [15]. Hwang et al. studied the removal of BTEX on an activated-carbon-coated electrode and the results showed that the removal performance was found in the following order: ethylbenzene > m-xylene > toluene > benzene. Moreover, the regeneration of the adsorbent required high heat and energy [16]. 

Recent advancements in the field have proven that transition-metal-containing catalysts are very effective in the removal of VOCs [1,4]. The physicochemical properties of the support nanomaterial play a significant role in the dispersion of metal oxides and thus their catalytic behavior [17]. Silica has been studied extensively as a support for catalysts wherein transition metal oxides play the catalytic role [15,18,19]. 

To consider the cost effectiveness of the process, it is required that the material be highly stable and retain its performance for multiple cycles. Several researchers performed different reusability tests. However, the literature lacks the fundamentals and a mechanism of how reusability relates to the chosen regeneration method. 

Therefore, in this study, two novel nanocomposites (Fe_2_O_3_/SiO_2_ and Fe_2_O_3_-Mn_2_O_3_/SiO_2_) have been fabricated for the first time using the co-precipitation/sol–gel method and have been compared for the removal of BTEX in ambient condition. UV power has been used to regenerate the synthesized nanocomposites, wherein these nanocomposites were used for BTEX adsorption for consecutive cycles to test their ability to be utilized in commercial applications. Properties of pristine and regenerated nanocomposites have been analyzed by Brunauer–Emmett–Teller (BET), X-ray powder diffraction (XRD), and Fourier-transform infrared spectroscopy (FTIR). Moreover, the mechanism related to the enhanced performance has been discussed in detail. 

## 2. Materials and Methods

### 2.1. Chemicals

Manganese(II) nitrate tetrahydrate Mn(NO_3_)_2_·4H_2_O (>98%) was supplied by Sigma Aldrich, Darmstadt, Germany. Ferric(III) nitrate nonahydrate (Fe(NO_3_)_3_·9H_2_O) was purchased from Samchun Pure Chemicals, Pyeongtaek-si, Republic of Korea. The hydrogen peroxide (28% H_2_O_2_) solution and sodium hydroxide (1 N NaOH), wee procured from Daejung Chemicals and Metals Co. Ltd., Siheung-si, Republic of Korea. Cetyltrimethylammonium chloride (CTAC) solution (25 wt.%), was procured from Sigma-Aldrich, Germany. Tetraethyl orthosilicate 95% (TEOS) was purchased from the Samchun Company, Pyeongtaek-si, Republic of Korea. All of the chemicals were used without any further purification.

### 2.2. Synthesis of the Nanocomposites 

The used catalyst in this study has been fabricated based on our previously published work with some modifications [20].

Synthesis of Fe_2_O_3_/Mn_2_O_3_ and Fe_2_O_3_ nanoparticles: First, Fe_2_O_3_/Mn_2_O_3_ was prepared by a sonication-assisted co-precipitation method. A total of 6 g of Fe(NO_3_)_3_·9H_2_O and 2.5 g of Mn(NO_3_)_2_·4H_2_O as precursors were dissolved in 300 mL of distilled water and kept at 65 °C while stirring them for 30 min. Then, 1 g cetyltrimethylammonium bromide (CTAB) as a surfactant was added to the stirring solution and mixed for 1 h at 65 °C. A precipitation agent (2.0 M NaOH) was added to the solution under sonication conditions until pH 12 was achieved. After 30 min, the sample was filtered and washed with distilled water, then dried at 100 °C overnight. The dried sample was calcined in a muffle furnace under air at 700 °C for 7 h. Fe_2_O_3_ was synthesized in the same way as above, but Mn(NO_3_)_2_·4H_2_O was not added during synthesis.

Synthesis of Fe_2_O_3_/SiO_2_ and Fe_2_O_3_-Mn_2_O_3_/SiO_2_: The sol–gel method was used to achieve the final product. First, the obtained nanoparticles (Fe_2_O_3_-Mn_2_O_3_ and Fe_2_O_3_) from the previous step were homogenized (PREMIX Model 2.5) for 8 h under 6000 rpm to prepare the nanoparticles for the next nanocomposite formation with SiO_2_. After homogenizing, 0.4 g of the nanoparticles were mixed with 40 mL of cetyltrimethylammonium chloride (CTAC) for 30 min at 40 °C. A total of 40 mL of TEOS was added to the solution, and a black color gel was made immediately. The obtained gel was incubated for 24 h under 50 °C with 200 rpm stirring and then calcinated for 6 h at 550 °C under air. The final product was coded as Fe_2_O_3_/SiO_2_ and Fe_2_O_3_-Mn_2_O_3_/SiO_2_.

### 2.3. Instruments 

For Brunauer–Emmett–Teller (BET) analysis, the N_2_ adsorption–desorption isotherm was measured at 77 K in a Gemini series Micromeritics 2360 instrument. Before subjecting for BET analysis, samples were degassed at 473 K for 2 h with a Micromeritics FlowPrep 060 (Norcross, GA, USA). The surface morphology of the nanocomposites was analyzed by scanning electron microscopy (SEM) (Hitachi S-4800, Tokyo, Japan). The finely grounded dried sample was coated with a gold–platinum alloy by ion-sputtering (E-1048 Hitachi ion sputter). The 2D elemental mapping was performed using energy-dispersive X-ray spectroscopy (EDS) (X-Maxn 80 T, Oxford, UK). Transmission electron microscope (TEM) images were collected over Philips CM200 (TEM), whereas 2D elemental mapping was performed using EDS DX-4 (EDAX). The diffraction pattern was obtained using a Rigaku D/Max-2500 X-ray diffractometer (XRD) with Cu K*α* and a Ni filter wherein the scanning speed was set to 3° min^−1^. A Fourier-transform infrared (FTIR) spectrum of the sample was recorded using KBr pellets over a PerkinElmer FTIR spectrometer (PerkinElmer, Waltham, MA, USA). The recording was done with a single-beam spectrometer with 60 added scans at a 0.02 m^−1^ resolution. The sample was dehydrated in the air at 673 K for 7 h. For X-ray photoelectron spectroscopic (XPS) analysis of samples, a Kratos Axis Ultra XPS instrument with a monochromatic Al K*α* X-ray source was used wherein the pressure was fixed to 1.33 × 10^−7^ Pa.

### 2.4. Experimental Methodology

GC set up: The VOCs gas used in the study was a 5 ppm concentration of the standard mixture gas of benzene, toluene, ethylbenzene, and xylene (BTEX). The concentration of the BTEX was analyzed by gas chromatography (Trace GC 1310, Thermo Fisher Scientific, Seoul, Republic of Korea). To prepare a calibration curve, high-purity nitrogen gas was used to dilute the gas and make various standard concentrations of BTEX. It should be noted that a 250 μL syringe was used for BTEX analysis. The used GC column in this study was TG-624 (30 m × 0.53 mm × 3.00 μm), and the initial temperature was programmed at 50 °C and increased by a ramp of 10 °C min^−1^ until it reached 120 °C, at which point the temperature remained constant for 1 min. The detector temperature was kept at 180 °C at the time of injection wherein the flow rate was 10 mL min^−1^.

**Figure 1:**
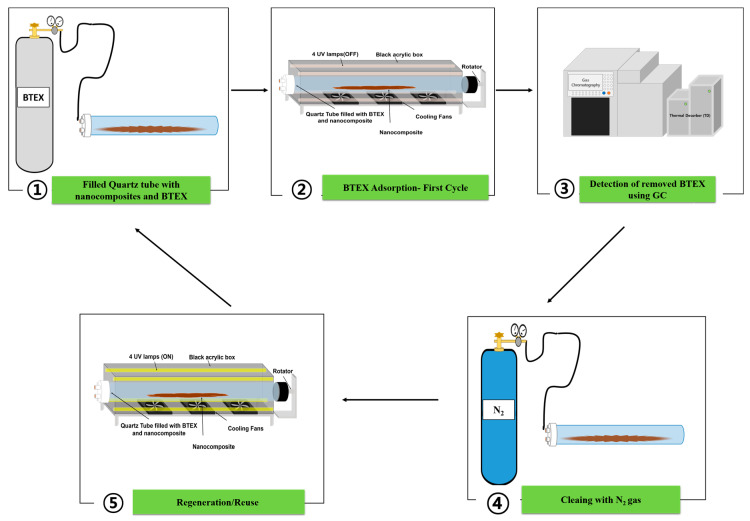
Schematic illustration of the experimental methodology.

Performance test: To test the removal performance, the nanocomposites were put into the 1L quartz tube, and the cell was filled with 1 L of BTEX. The tube filled with BTEX was continuously rotated inside the reactor (Figure 1), and the gas samples were taken using the syringe at specific time intervals. The experiments were conducted using two nanocomposites (Fe_2_O_3_/SiO_2_ and Fe_2_O_3_-Mn_2_O_3_/SiO_2_) with different dosages of (0.05, 0.1, 0.5, 1, and 2 g), and the optimism dosage was chosen to do the regeneration tests. The syringe and quartz tube were washed with nitrogen and conditioned twice before use. 

Regeneration test: The regeneration test was performed in the same reactor with the same condition. However, the cycle test was carried out, followed by 1 h of UV (19 W, UV-C, I_max_ ~254 nm, Philips, Amsterdam, Netherlands) irradiation of the nanocomposites. After BTEX adsorption cycle, N_2_ gas was sufficiently injected into the tube so that all of the BTEX gas remaining in the tube was washed away. After that, the nanocomposites was exposed to UV irradiation for regeneration for 1 h, and the adsorption–regeneration process was performed in the same way for the next cycle. The regeneration process was repeated 10 times to evaluate the efficiency of nanocomposites for BTEX removal. The removal efficiency of BTEX was calculated using the following equation, where (C_i_) is the BTEX concentration at specific time intervals and (C_o_) is the initial concentration of BTEX.

(1) $$\mathrm{BTEX}\;\mathrm{removal}\;\left(\%\right)=\left(1-\frac{C_{i}}{C_{o}}\right)\times 100$$

## 3. Results and Discussions

### 3.1. Pristine Catalyst 

**Figure 2:**
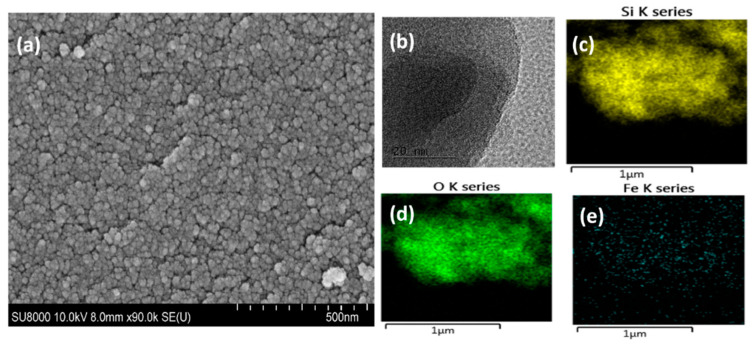
(**a**) SEM, (**b**) TEM, (**c**–**e**) elemental spectrum of Fe_2_O_3_/SiO_2_.

**Figure 3:**
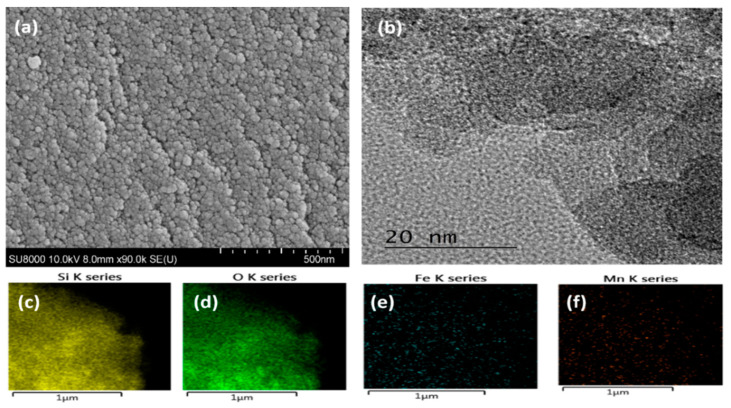
(**a**) SEM, (**b**) TEM, (**c**–**f**) elemental spectrum of Fe_2_O_3_-Mn_2_O_3_/SiO_2_.

Morphology of the pristine nanocomposite: To evaluate the physicochemical properties of the synthesized catalyst, field-emission scanning electron microscopy (FE-SEM) and high-resolution transmission electron microscope (HR-TEM) were used. The FE-SEM images showed a porous surface with a spheroid-like morphology for Fe_2_O_3_/SiO_2_ nanocomposites (Figure 2). The HR-TEM images were taken with a field emission TEM and Fe_2_O_3_/SiO_2_. Fe metal oxide particles were dispersed in the silica matrices (Figure 2b). Furthermore, 2D mapping using energy-dispersive X-ray spectroscopy (EDX) showed that the Fe_2_O_3_/SiO_2_ nanocomposite consists of uniformly distributed Si, Fe, and O (Figure 2c–e). For Fe_2_O_3_-Mn_2_O_3_/SiO_2_, similar results have been shown, and the 2D mapping of Fe overlaps with that of the Mn, which indicates the vicinity of Mn_2_O_3_ and Fe_2_O_3_ nanoparticles where both elements are uniformly distributed silica matrices (Figure 3a–f). 

**Figure 4:**
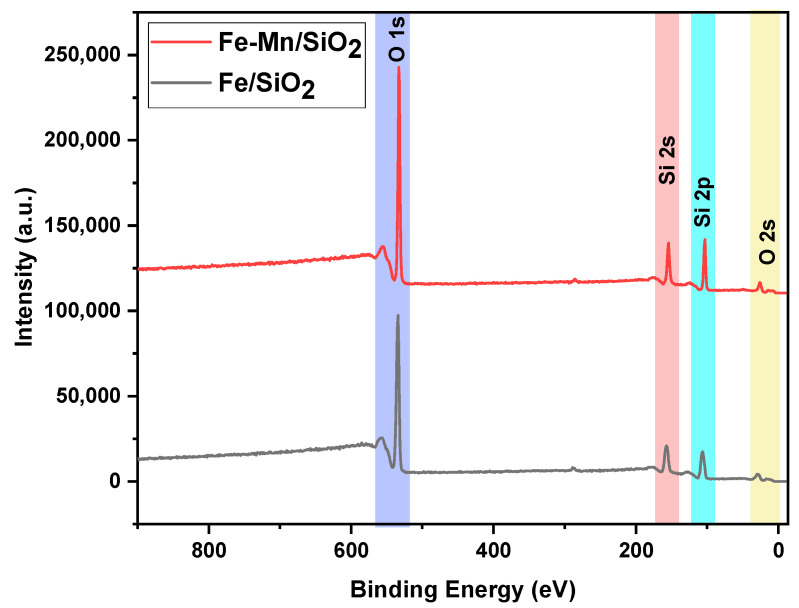
HRXPS full spectrum of nanocomposites.

HRXPS: In order to investigate the formation of nanocomposites, existing elements in the catalyst were additionally tested using X-ray photoelectron spectroscopy (XPS) analysis. High-energy resolution scans of Fe_2_O_3_/SiO_2_ showed the existence of the Si2p, Fe2p, and O1s in the nanocomposites, and Fe_2_O_3_-Mn_2_O_3_/SiO_2_ (Fe-Mn/SiO_2_) was also detected (Figure 4). It should be noted that the concentration of Fe and Mn is low, which results in the absence of peaks in full spectra in Figure 4. Table 1 showed the atomic ration of elements in both nanocomposites. Our previous study was on the removal of dye with Fe-Mn/SiO_2_, which is same nanocomposite in the current study [20]. The XPS results showed that Fe existed in the form of Fe^3+^ and that Mn stayed in the form of Mn^3+^. Even after UV irradiation on the nanocomposite, the state of Fe and Mn remained unaffected by UV, indicating that there was no significant change in the XPS spectra. 

### 3.2. Catalyst Dosage 

**Figure 5:**
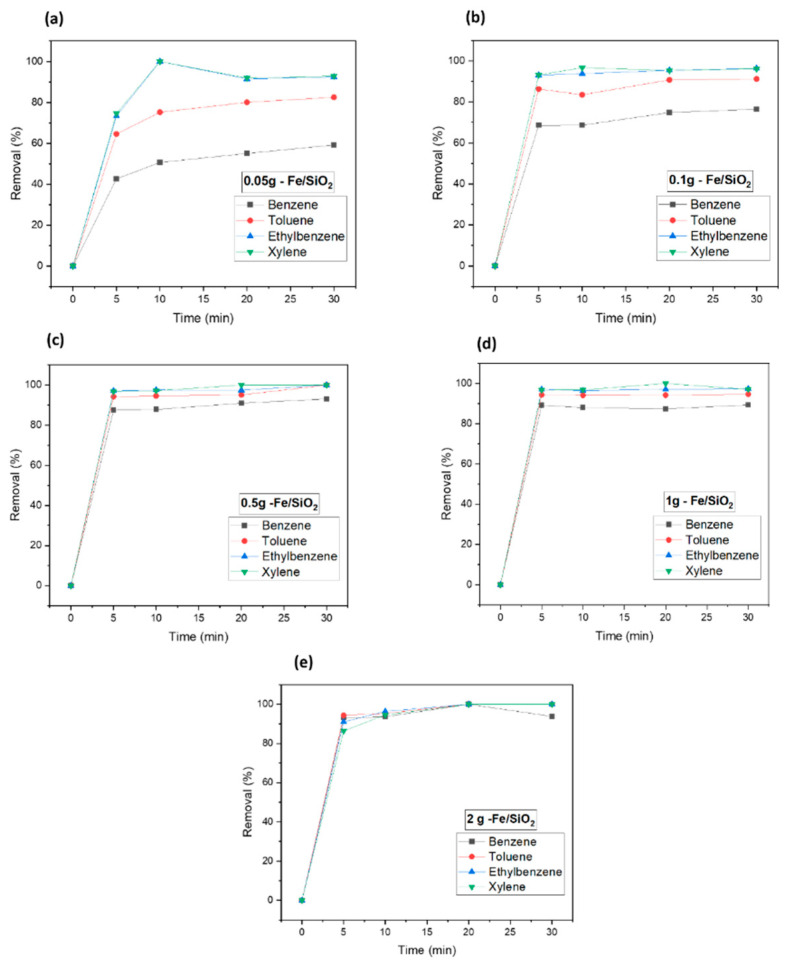
Catalyst dosage experiment for Fe/SiO_2_ (Fe_2_O_3_/SiO_2_) of nanocomposites. (**a**) 0.05 g—Fe/SiO_2_, (**b**) 0.1 g—Fe/SiO_2_, (**c**) 0.5 g—Fe/SiO_2_, (**d**) 1 g—Fe/SiO_2_, (**e**) 2 g—Fe/SiO_2_.

**Figure 6:**
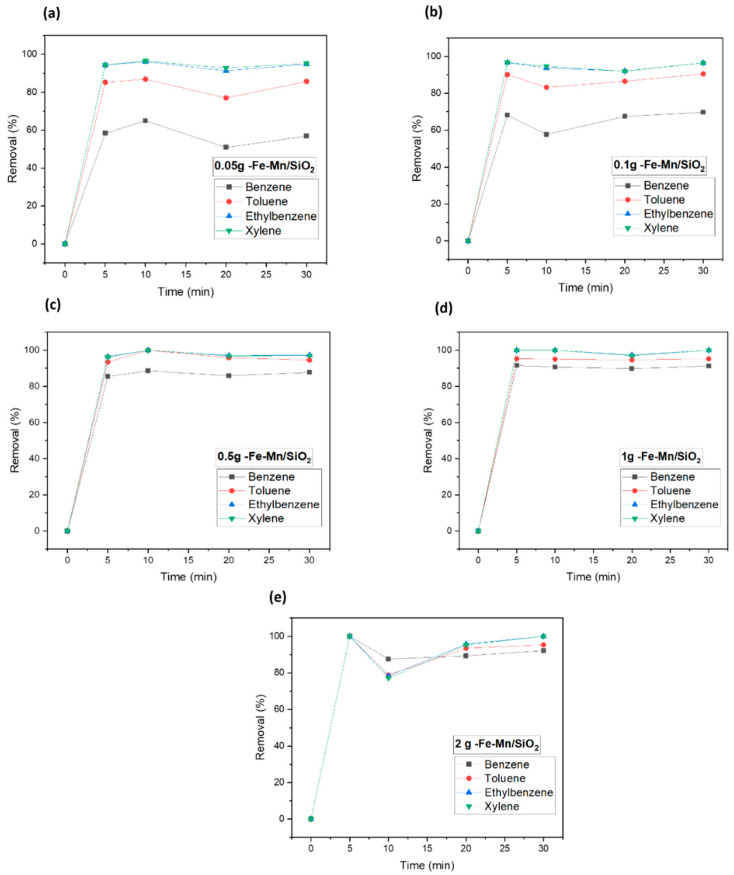
Catalyst dosage experiment for Fe-Mn/SiO_2_ (Fe_2_O_3_-Mn_2_O_3_/SiO_2_) of nanocomposites. (**a**) 0.05 g—Fe/SiO_2_, (**b**) 0.1 g—Fe/SiO_2_, (**c**) 0.5 g—Fe/SiO_2_, (**d**) 1 g—Fe/SiO_2_, (**e**) 2 g—Fe/SiO_2_.

In order to study the effect of catalyst dosage on the degradation of VOCs, adsorption experiments were performed with different dosages (0.05, 0.1, 0.5, 1, and 2 g). Thus, the effect of reaction time for BTEX removal was studied for 5, 10, 20, and 30 min. As shown in Figure 5a, the removal efficiency of each BTEX increased with increasing reaction time. The removal efficiency of benzene in the BTEX mixture increased from 42% at 5 min to 59% at 30 min. However, as the dosage of Fe_2_O_3_/SiO_2_ increased, about 90% of the benzene and 97% of ethylbenzene were removed within 5 min. For the Fe_2_O_3_-Mn_2_O_3_/SiO_2_ catalyst, the removal efficiency also increased as the catalyst dosage increased (Figure 6). Thus, the removal efficiency for benzene was 87% but more than 94% for the other three gases. The benzene removal efficiency with Fe_2_O_3_-Mn_2_O_3_/SiO_2_ increased from 69% to 87% when the dosage increased from 0.1 g to 0.5 g. While ethylbenzene and xylene showed 100% removal with both catalysts (2 g L^−1^ catalyst dosage at 20 min), benzene showed 92–93% removal efficiency, which was the lowest adsorption capacity among the BTEX component. The interaction between BTEX and catalyst surface is contributed to by van der Waals’ force, which is dependent on the molecular weight (78.12 g mol^−1^ for benzene, 92.15 g mol^−1^ for toluene, and 106.17 g mol^−1^ for ethylbenzene and xylene) [21,22]. The higher the molecular weight, the more the adsorption capacity increases. Konggidinata et al. conducted the BTEX adsorption on mesoporous carbon and found the adsorption capacity to follow the order of xylenes > ethylbenzene > toluene > benzene due to the different solubility of gases [22]. Areerob et al. carried the adsorption performance of BTEX with silica-based material, and the performance was proportional to their hydrophobicity and molecular weight [21]. Thus, the interaction between BTEX and catalyst surface is contributed by van der Waals’ force, which is dependent on the molecular weight (78.12 g mol^−1^ for benzene, 92.15 g mol^−1^ for toluene, and 106.17 g mol^−1^ for ethylbenzene and xylene) [21,22]. The higher the molecular weight, the more the adsorption capacity increases. Given that the adsorption of benzene is the least adsorbed among the four gases due to its water solubility and molecular weight; further experimental parameters for catalyst dosage were chosen based on the results of benzene removal efficiency (0.5 g L^−1^ of catalyst dosage). The results of this study has been compared with similar works and shown in Table 2.

### 3.3. Performance Test

**Figure 7:**
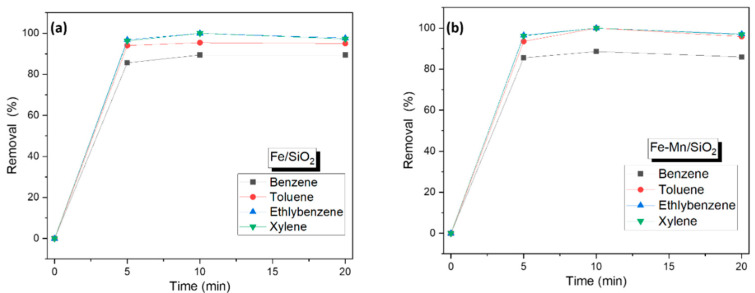
BTEX removal using an optimized amount of nanocomposites (**a**) Fe/SiO_2_ (Fe_2_O_3_/SiO_2_) (**b**) Fe-Mn/SiO_2_ (Fe_2_O_3_-Mn_2_O_3_/SiO_2_).

The performance of the nanocomposites has been tested and compared for the removal of 5 ppm of BTEX. The results showed a similar removal performance for both nanocomposites with the following order of ethylbenzene > m-xylene > toluene > benzene (Figure 7). The benzene removal efficiency was lower compared to other gases, as described above due to having a lower molecular weight. The primary mechanism supporting the adsorption of a BTEX mixture on SiO_2_ surfaces is van der Waals’ force. The larger the molecule, the higher the contribution of van der Waals’ force to the adsorption potential. In the four mixtures, it can be said that ethylbenzene (106.20 g mol^−1^) and xylene (106.20 g mol^−1^), which have the largest molecules, exhibit the best adsorption potential. The molecule of the toluene (92 g mol^−1^) has a relatively lower adsorption potential than the molecules of either ethylbenzene or xylene. Benzene (78.10 g mol^−1^) has the smallest molecule among the four gas mixtures, which clearly indicates the low adsorption efficiency of SiO_2_. The surface area of the van der Waals’ of BTEX gas are 110 Å^2^, 132 Å^2^, 159 Å^2^, and 159 Å^2^, respectively, which shows that they are quite proportional to the adsorption capacity of SiO_2_. In this study, since four mixed gases were used to examine the adsorption performance of SiO_2_, the comparative difference in molecular weight between the four mixed gases is very large. In addition, the functional group of adsorbents is a significant factor in the adsorption process, and in general, SiO_2_ does not have many functional groups. Therefore, it does not significantly induce nonspecific attraction in micro-pores such as van der Waals’ interaction. Another key characteristic of an adsorbent relative to its adsorption capacity is the K_OW_ value, which indicates solubility. However, SiO_2_ is a relatively hydrophilic adsorbent compared to the carbon-based adsorbent. Therefore, the K_OW_ is not a significant factor in the adsorption mechanism. 

### 3.4. Regeneration/Reuse 

**Figure 8:**
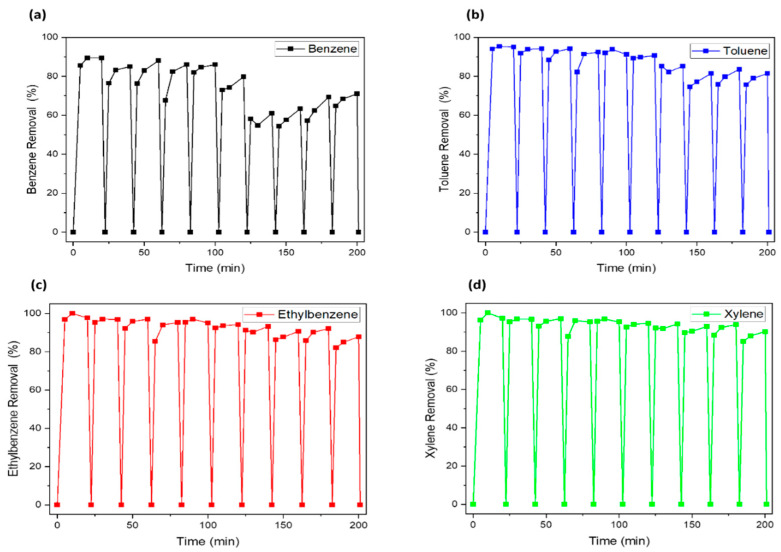
BTEX removal by UV–Fe_2_O_3_/SiO_2_: (**a**) benzene, (**b**) toluene, (**c**) ethylbenzene, (**d**) xylene.

**Figure 9:**
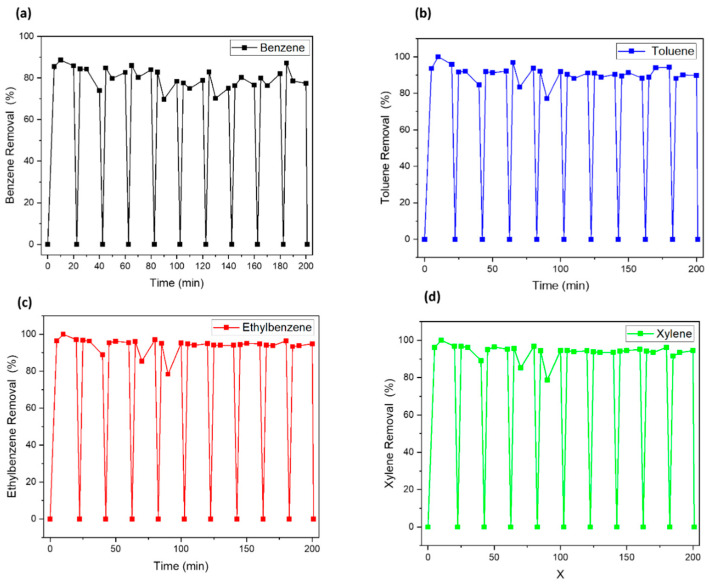
BTEX removal by UV–Fe_2_O_3_-Mn_2_O_3_/SiO_2_: (**a**) benzene, (**b**) toluene, (**c**) ethylbenzene, (**d**) xylene.

The removal experiments were repeated in the same condition as the first cycle following the regeneration of the used nanocomposite with UV (Figure 8 and Figure 9). Similar regeneration methods have been used in our previously published paper [29]. Both nanocomposites showed good reusability, revealing that the UV irradiation process is suitable for the regeneration method for metal-doped porous silica material. Both adsorbents maintained stable adsorption efficiency in repeated experiments ten or more times. However, in the case of benzene removal by using Fe-SiO_2_, a slight decrease in removal efficiency was observed on the sixth occasion (Figure 8). The removal efficiency was 61% at the seventh cycle and 71% at the tenth cycle. This is considered to be because the pore structure of the Fe-SiO_2_ material is changed to a more suitable structure for removing contaminants having a relatively larger molecular weight, as the average pore size is changed in width by long-term UV irradiation [30]. Nevertheless, the UV irradiation used in this study effectively mineralized the adsorbed contaminants, so it can be explained that the removal efficiency is continuously maintained. The regeneration mechanism by UV irradiation is mainly photo-fenton reactions between the irradiated UV and the doped metal source in the SiO_2_. Thus, it can be seen that the regeneration is more efficient because the moisture adsorbed to silica with high hygroscopic behavior can play a role as a source of OH radicals. This can show that regeneration by UV irradiation is an effective method for photo-catalyst adsorbent reuse in this study. In the next chapter, the change in the physicochemical structure of materials will be discussed by a comparison of pristine and UV-regenerated catalyst. 

### 3.5. Comparison of Pristine and Regenerated Catalyst

**Figure 10:**
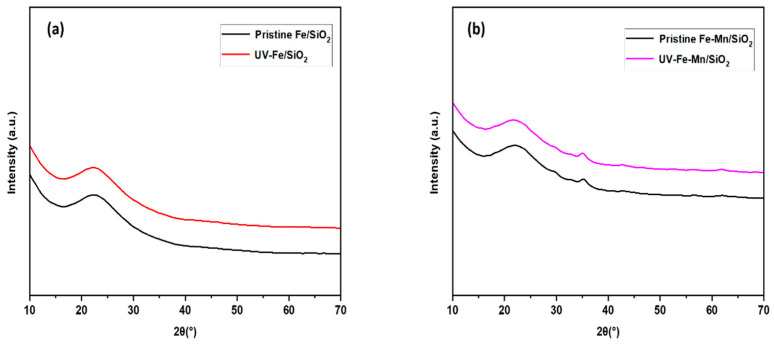
(**a**) XRD patterns (**a**) pristine and UV-irradiated Fe_2_O_3_/SiO_2_ (Fe/SiO_2_) (**b**) Fe_2_O_3_-Mn_2_O_3_/SiO_2_ (Fe-Mn/SiO_2_).

XRD: X-ray diffraction (XRD) was analyzed to determine the phase identification of the synthesized materials. The change in the XRD patterns of Fe_2_O_3_/SiO_2_ (Fe/SiO_2_) and Fe_2_O_3_-Mn_2_O_3_/SiO_2_ (Fe-Mn/SiO_2_) before and after UV irradiation are shown in Figure 10. The XRD spectrum of all samples showed a broad diffraction peak at 22.4°, which indicates a typical amorphous SiO_2_ network [31]. This indicates that Fe or Fe-Mn nanoparticles were incorporated into the SiO_2_. For both Fe_2_O_3_/SiO_2_ (Fe/SiO_2_) and Fe_2_O_3_-Mn_2_O_3_/SiO_2_ (Fe-Mn/SiO_2_), there are no significant peaks for Fe due to the low synthetic content (under 1%). Based on our previous study, the XRD pattern of Fe-Mn nanoparticles (without a SiO_2_ frame) matched the rhombohedral α-Fe_2_O_3_ (JCPDS #33-0664) and cubic α-Mn_2_O_3_ (JCPDS #24-0508) peaks [32]. However, Fe_2_O_3_-Mn_2_O_3_/SiO_2_ (Fe-Mn/SiO_2_) contains a mixture of amorphous and slight crystal phases in the diffraction at 2θ of 35.4°, which is identified as Mn_2_O_3_ (JCPDS No. 24-508). There was no significant change in the XRD spectrum of both Fe_2_O_3_/SiO_2_ (Fe/SiO_2_) and Fe_2_O_3_-Mn_2_O_3_/SiO_2_ (Fe-Mn/SiO_2_) before and after UV. This indicates that the UV exposure did not cause structure change or crystal formation, given that there is no change in the XRD pattern depending on whether or not there was UV exposure.

**Figure 11:**
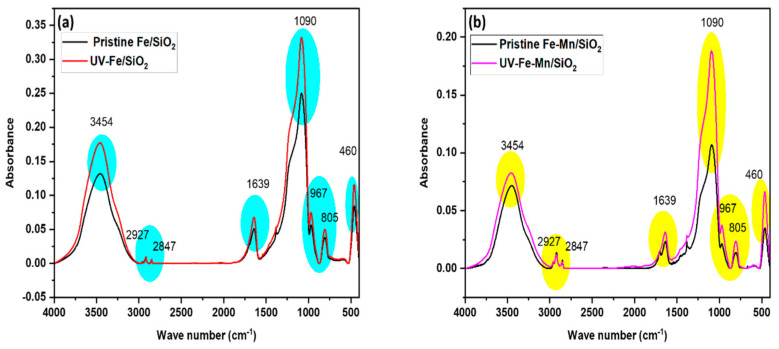
FTIR spectrum (**a**) pristine and UV−irradiated Fe_2_O_3_/SiO_2_ (Fe/SiO_2_) (**b**) Fe_2_O_3_−Mn_2_O_3_/SiO_2_ (Fe−Mn/SiO_2_).

FTIR: Fourier-transform infrared spectroscopy (FTIR) was introduced to detect different functional groups in synthesized materials in the range of 400 to 4000 cm^−1^. The change in FTIR absorbance of Fe_2_O_3_/SiO_2_ (Fe/SiO_2_) and Fe_2_O_3_-Mn_2_O_3_/SiO_2_ (Fe-Mn/SiO_2_) and after UV irradiation is shown in Figure 11. All samples showed similar peak locations with different absorbance intensities depending on UV exposure. It can be seen that several bands for the SiO_2_ network at around 1090 cm^−1^ for the antisymmetric stretching of Si-O-Si, 967 cm^−1^ for the stretching vibration of Si-OH, 805 cm^−1^ for the symmetric mode of Si-O-Si, and 460 cm^−1^ for Si-O [33,34]. The band at around 3454 cm^−1^ is concomitant with the Si-OH of surface hydroxyl groups, and the wave number at 1639 cm^−1^ is characterized by the -OH group, which commonly exists in water molecules [35]. The band at 460 cm^−1^ is allocated to the Si-O and M-O bond (metal oxides) in the MO_6_ octahedron and MO_4_ tetrahedron at the same time. Given the Si-O bond presence in all synthesized materials, the band for metals impregnated in SiO_2_ can be hidden due to overlapping with Si-O [20]. The band at 2847 cm^−1^ and 2927 cm^−1^ can be assigned to the C-H stretching due to the CTAB surfactant, which was used as a synthetic material [36]. 

The UV-irradiated Fe_2_O_3_/SiO_2_ (Fe/SiO_2_) and Fe_2_O_3_-Mn_2_O_3_/SiO_2_ (Fe-Mn/SiO_2_) resembled that of pristine materials but with increased absorbance. The increased intensity at 967 cm^−1^ (Si-OH) is due to the formation of new hydroxyl groups, and it can be verified by the increased intensity of the 3454 cm^−1^. 

Based on these results, it seems that the existing Si–O–Si bonds may have broken and been rearranged after UV irradiation, and a SiO_2_ network with a higher content of OH groups has been formed, which can be one of the reasons for the high performance and reusability of both nanocomposites. In the adsorption process, these increased OH groups increase the number of hydrogen bonds between the oxygen of silanol groups with hydrogen molecules on BTEX and enhance the removal [29]. It should be noted that this finally brings more BTEX molecules into the nanocomposites’ vicinity and improves the possibility of degradation along with adsorption. 

**Figure 12:**
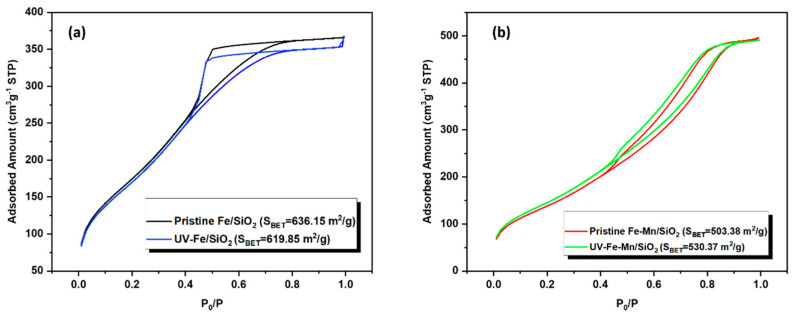
N_2_ adsorption–desorption curve (**a**) pristine and UV−irradiated Fe_2_O_3_/SiO_2_ (Fe/SiO_2_) (**b**) Fe_2_O_3_−Mn_2_O_3_/SiO_2_ (Fe−Mn/SiO_2_).

BET: The porosity properties and specific surface area of pristine and UV irradiated nanocomposites were analyzed by N_2_ adsorption–desorption isotherm (Figure 12). As shown in the figure, the sorption isotherm of both nanocomposites was type IV, which belongs to the structure of mesoporous materials. Based on the Brunauer-Emmett-Teller method, pristine Fe_2_O_3_-Mn_2_O_3_/SiO_2_ (Fe-Mn/SiO_2_) has a specific surface area (*S*_BET_) of 503.38 m^2^ g^−1^, which increased to 530.37 after UV irradiation. However, the sample without Mn surface area did not increase. 

### 3.6. Mechanism of the Regeneration Performance 

Adsorption: The primary mechanism supporting the adsorption of a BTEX mixture on SiO_2_ surfaces is van der Waals’ force. Based on BET results, the surface area of Fe_2_O_3_-Mn_2_O_3_/SiO_2_ increases after UV regeneration, which can increase the chance of adsorption. Based on FTIR results, OH group density increases, which can increase the number of hydrogen bonds between the oxygen of silanol groups and the hydrogen in BTEX molecules. It should be noted that this can induce more BTEX molecules in the vicinity of the nanocomposites and increases the chance of degradation along with adsorption. 

**Figure 13:**
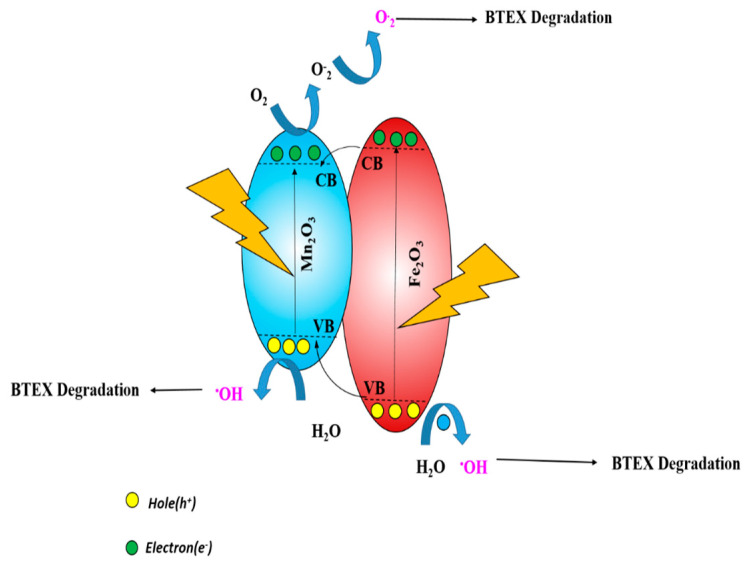
The degradation mechanism of BTEX on the surface of Fe_2_O_3_-Mn_2_O_3_/SiO_2_.

Degradation of the adsorbed BTEX on the surface: Even though in the first cycle the removal performance of two nanocomposites was similar, after performing the reusability test, Fe_2_O_3_-Mn_2_O_3_/SiO_2_ was found to be more effective for long-term use, and the increased performance can be attributed to the effect of Mn, which can increase the chances of pollutant degradation under UV irradiation. The mechanism is shown in Equations (2)–(6). When UV radiations interact with the surface of the Fe_2_O_3_-Mn_2_O_3_/SiO_2_, e^−^/h^+^ pairs are generated in both Fe_2_O_3_ and Mn_2_O_3_ due to electron transfer from the valence band to the conduction band [20]. Under UV light irradiation, the electrons from the Fe_2_O_3_ transfer to the conduction bands of Mn_2_O_3_, increasing their reduction potential [37]. This will prevent the recombination of the hole and the electron pair, thereby increasing their life enough to generate a chain of reactions to degrade the BTEX adsorbed in the surface. The Fe_2_O_3_ hole will go into the valance band of the Mn_2_O_3_, raising its oxidation potential. This inhibits its electron/hole pair from combining and releasing energy. Holes h^+^ on the degradation process where h^+^ can induce a reaction to produce OH radicals and finally degrade the attached organic pollutants attach to the surface of the catalyst and clean the catalyst surface. The e^−^ also can start another reaction to produce more active OH radicals using the oxygen in the air (Equations (7) and (8)) [38] This eventually can make the catalyst highly reusable and economical. It should be noted that, when Mn exists in the structure, more h^+^ can be generated. Consequently, the Fe_2_O_3_-Mn_2_O_3_/SiO_2_ could retain the performance for a longer time compared to Fe_2_O_3_/SiO_2._ The explained mechanism has been shown in Figure 13.

(2) $${\mathrm{Fe}}_{2}O_{3}+\mathrm{hv}\to {\mathrm{Fe}}_{2}O_{3}^{*}(e_{\mathrm{CB}}^{-}+h_{\mathrm{VB}}^{+})$$

(3) $${\mathrm{Mn}}_{2}O_{3}+\mathrm{hv}\to {\mathrm{Mn}}_{2}O_{3}^{*}(e_{\mathrm{CB}}^{-}+h_{\mathrm{VB}}^{+})$$

(4) $$h_{\mathrm{VB}}^{+}+\mathrm{OBTEX}\to \mathrm{degradation}\;\mathrm{of}\;\mathrm{BTEX}$$

(5) H2O+hVB+→O•H+ H+

(6) O•H+BTEX→degradation of BTEX

(7) $$O_{2}+e_{\mathrm{CB}}^{-}\to O_{2}^{-}$$

(8) H2O+O2−→O•H+ H+

## 4. Conclusions

In this study, two novel nanocomposite materials (Fe_2_O_3_/SiO_2_ and Fe_2_O_3_-Mn_2_O_3_/SiO_2_) were fabricated using two-step co-precipitation and the sol–gel method, then compared the results for their ability to remove BTEX. The nanocomposites had been regenerated using UV power to test their reusability. This research revealed that in the first cycle, both nanocomposites could remove more than 85% of the BTEX. The reusability test showed that Fe_2_O_3_-Mn_2_O_3_/SiO_2_ could retain its performance for ten consecutive cycles. This enhanced performance could be attributed to structural variations after UV irradiation, which induces more BTEX adsorption. Results showed that, after UV irradiation, OH group density on the nanocomposites increases, which finally can increase the possibility of hydrogen bonds between nanocomposites and BTEX molecules. Moreover, the surface area was also increased after UV irradiation and increased the number of active sites. Additionally, adsorption and degradation could occur simultaneously since the adsorbed BTEX on the catalyst surface could be degraded by the UV irradiation process, which could finally clean the surface of the catalyst and make it ready for the next cycles. It should be noted that both Fe and Mn can produce h^+^ under UV irradiation and that this h^+^ can be a reason to degrade organic pollutants. However, among benzene, toluene, ethylbenzene, and xylene, the removal efficiency of benzene stayed around 80%, while the removal efficiency of the other three VOCs maintained almost 100% removal. Further works should develop the removal performance on benzene and evaluate the performance of this in practical conditions. Thus, the adsorption sensibility should be carried out on a specific VOC contaminant. 

## Figures and Tables

**Table 1:** Atomic ratio of elements used in the synthesis of nanocomposites.

Element/Nanocomposites	Atomic% (Fe_2_O_3_/SiO_2_)	Atomic% (Fe_2_O_3_-Mn_2_O_3_/SiO_2_)
O1s	63.08	65.33
C1s	5.13	2.79
Si2p	31.1	30.71
Fe2p	0.68	0.7
Mn2p	-	0.48

**Table 2:** Comparison of VOCs removal performance by the nanomaterials used in this study with other nanomaterials.

Nanomaterial	VOC	Performance	Ref.
TiO_2_/AC	Formaldehyde 1 ppm	33.9%	[23]
MnO_2_/MWCNT	Formaldehyde 10 ppm	43%	[24]
P25/graphene	Benzene 156 ppm	8%	[25]
CNTs/TiO_2_	Limonene 1.6 ppm	42%	[26]
10 wt.% CoO*_x_*/Al_2_O_3_	Acetone 200 ppm	75%	[27]
10% SiO_2_/TiO_2_ fiber	Toluene 7 ppm	90.6%	[28]
Fe_2_O_3_-Mn_2_O_3_/SiO_2_	Ethylbenzene 5 ppm	97.07%	This study
Fe_2_O_3_-Mn_2_O_3_/SiO_2_	m-xylene ppm	96.85%	This study
Fe_2_O_3_-Mn_2_O_3_/SiO_2_	Toluene ppm	95.82%	This study
Fe_2_O_3_-Mn_2_O_3_/SiO_2_	Benzene ppm	85.89%	This study
